# Supplementary material for: Inhibition of de novo ceramide biosynthesis affects aging phenotype in an in vitro model of neuronal senescence
Source: Aging (Albany NY). 2019 Aug 29;11(16):6336–57. doi: 10.18632/aging.102191 (PMC6738398; doi:10.18632/aging.102191)
Supplement: Supplementary Figures [file aging-11-102191-s002.pdf]

SUPPLEMENTARY FIGURES

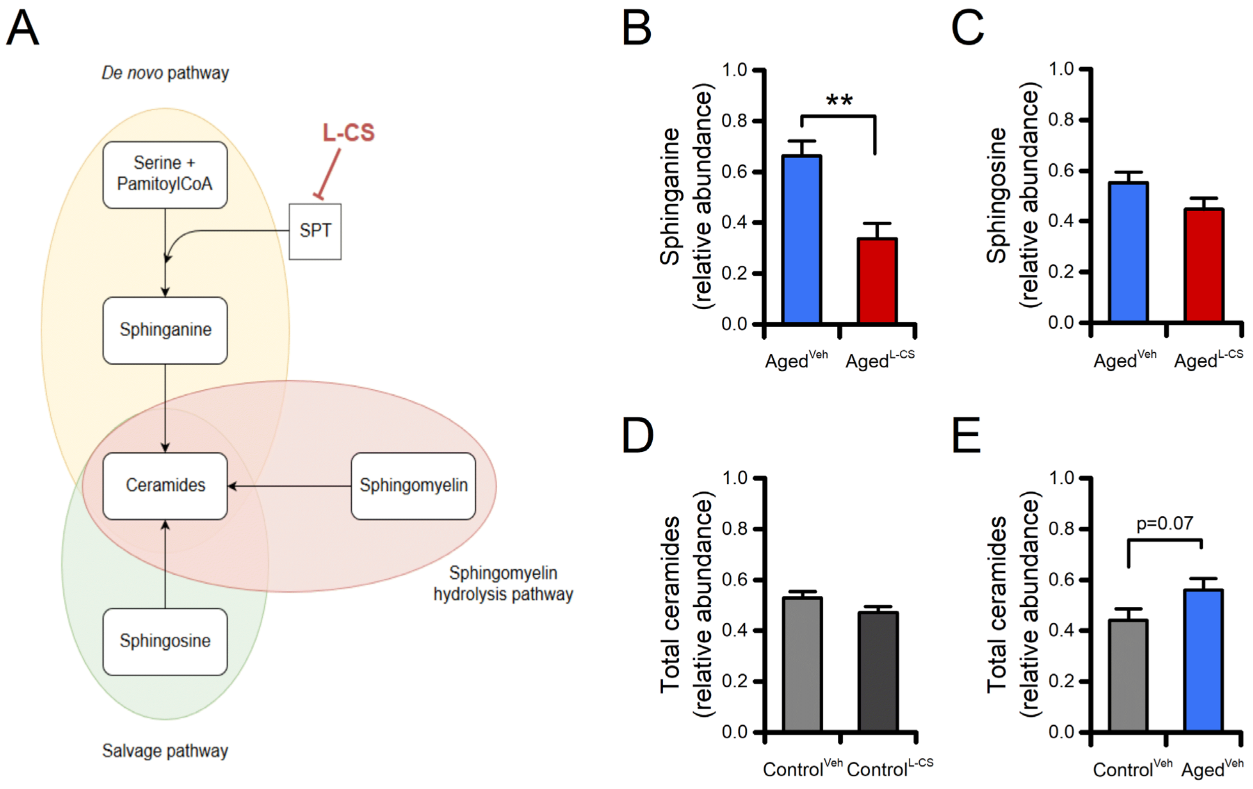

Supplementary Figure 1. Effects of aging and L-CS on *de novo* ceramide biosynthesis. (**A**) The pictogram illustrates a simplified version of the steps involved in the three ceramide biosynthetic pathways. Please, note that L-CS acts by inhibiting SPT in the *de novo* pathway. (**B**) Bar graphs depict the relative abundance of sphinganine in vehicle- and L-CS-treated aged neurons (n=3). (**C**) Bar graphs depict the relative abundance of sphingosine in vehicle- and L-CS-treated aged neurons (n=3). (**D**) Bar graphs depict the relative abundance of ceramides in vehicle- and L-CS-treated control neurons (n=3). (**E**) Bar graphs depict the relative abundance of ceramides in vehicle-treated control and aged neurons (n=3). Means were compared by unpaired Student t-test. ** indicates p<0.01.

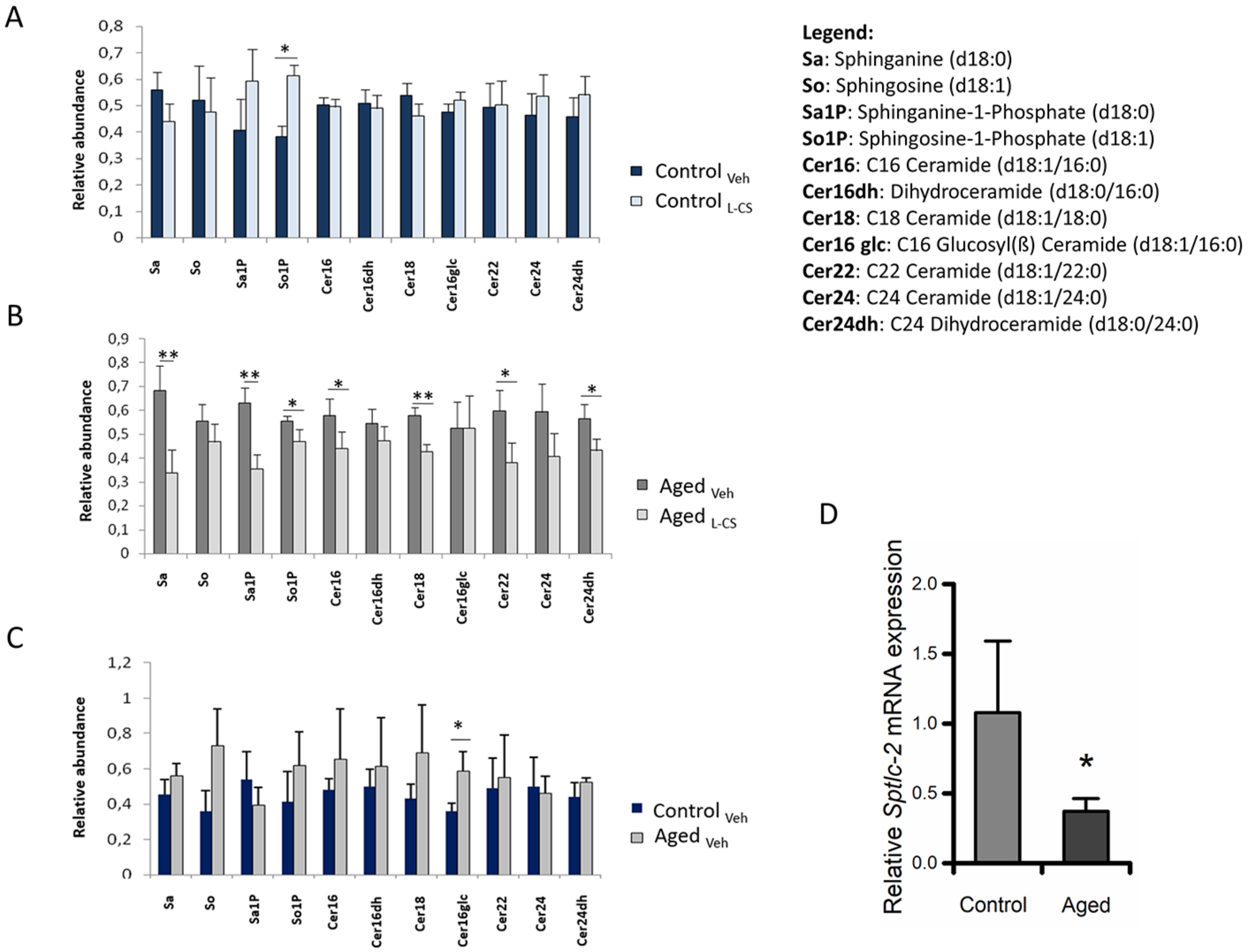

Supplementary Figure 2. Effects of aging and L-CS on *de novo* ceramide biosynthesis. (A) Bar graphs depict the relative abundance of the molecules as measured by LC-MS/MS in vehicle- and L-CS-treated control neurons (n=3). (**B**) Bar graphs depict the relative abundance of the molecules as measured by LC-MS/MS in vehicle- and L-CS-treated aged neurons (n=3). (**C**) Bar graphs depict the relative abundance of the molecules as measured by LC-MS/MS in vehicle-treated control and aged neurons (n=3). (**D**) Bar graphs depict mRNA levels of *Sptlc-2* measured by real-time PCR in control and aged cultures (n=3-5 per condition). Means were compared by unpaired Student t-test. * indicates p<0.05, ** indicates p<0.01.

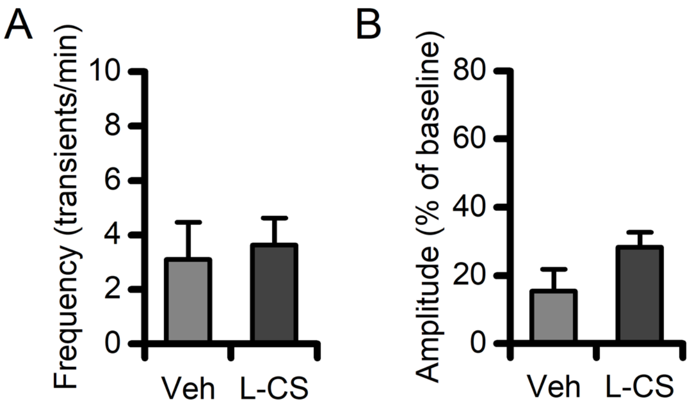

Supplementary Figure 3. Effects of L-CS on dendritic Ca^{2+} transient in control cultures. (**A**) Bar graphs depict average transient frequencies of $Control^{Veh}$ and $Control^{L\text{-}CS}$ dendrites ($Control^{Veh}$ n=11 and $Control^{L\text{-}CS}$ n=14 dendrites from 6-7 experiments). (**B**) Bar graphs depict the average dendritic Ca^{2+} transient amplitude in the two study groups [samples are the same as in (**B**)]. Means were compared by unpaired Student t-test.

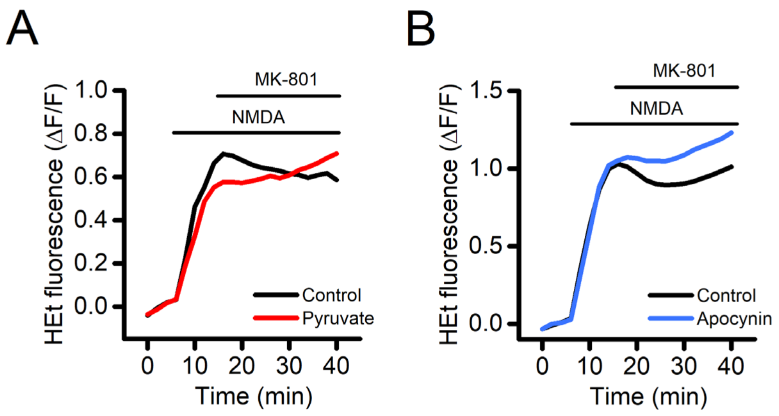

Supplementary Figure 4. Mitochondria are the primary source of ROS in our cortical cultures. To evaluate the primary source of ROS in our system we performed pharmacological manipulations aimed at selectively observing ROS of mitochondrial and non-mitochondrial origin. HEt-loaded control cortical neurons were challenged with NMDA + glycine (50 µM + 10 µM), a maneuver that triggers a robust generation of ROS from both mitochondrial and non-mitochondrial sources [80,81]. After 5 minutes, NMDA receptor (NMDAR) overactivation was halted by bath application of the non-competitive NMDAR antagonist MK-801 (10 µM). In (**A**), traces depict NMDA-driven ROS generation in control neurons (black trace) and in cells bathed and challenged in a solution in which glucose was replaced with pyruvate (15 mM), an established paradigm aimed at promoting ROS generation only from mitochondria. No differences were observed between the two conditions. In (**B**), neurons were challenged with (blue trace) or without (black trace) apocynin (500 µM), an inhibitor of NADPH oxidase activity. Means were compared by unpaired Student t-test. No differences were observed between the two conditions. Collectively, these findings indicate that mitochondria are the primary source of ROS in our cortical cultures.
