# Supplementary material for: Inhibition of de novo ceramide biosynthesis affects aging phenotype in an in vitro model of neuronal senescence
Source: Aging (Albany NY). 2019 Aug 29;11(16):6336–57. doi: 10.18632/aging.102191 (PMC6738398; doi:10.18632/aging.102191)
Supplement: Supplementary Table 1 [file aging-11-102191-s001.pdf]

SUPPLEMENTARY TABLE

Supplementary Table 1. Data related to mitochondrial morphology analysis as shown in Fig. 3.

	**Control^{Veh}**	**Control$^{L\text{-}CS}$**	**Aged^{Veh}**	**Aged$^{L\text{-}CS}$**	**P**
**Individuals (*n*)**	191.00 ± 46.27	168.4 ± 34.11	251.00 ± 33.49	217.80 ± 54.14	>0.05
**Networks (*n*)**	64.25 ± 16.28	38.6 ± 11.69	78.16 ± 9.81	71.2 ± 15.61	>0.05
**Mean Branch Length (µm)**	0.602 ± 0.020	0.579 ± 0.012	0.560 ± 0.016	0.547 ± 0.010	>0.05
**Median Branch Length (µm)**	0.456 ± 0.014	0.467 ± 0.013	0.435 ± 0.014	0.417 ± 0.007	>0.05
**Length Standard Deviation (µm)**	0.531 ± 0.035	0.464 ± 0.015	0.486 ± 0.017	0.490 ± 0.008	>0.05
**Median Network Size (*n* of branches)**	4.25 ± 0.47	4.40 ± 0.40	4.41 ± 0.55	4.10 ± 0.45	>0.05
**Mitochondrial Footprint (µm^2)**	139.19 ± 30.06	241.26 ± 47.43	189.39 ± 27.35	153.51 ± 24.35	>0.05
